# Machine learning algorithm of ultrasound-mediated intestinal function recovery and nursing efficacy analysis of lower gastrointestinal malignant tumor after surgery

**DOI:** 10.12669/pjms.37.6-WIT.4866

**Published:** 2021

**Authors:** Lei Ma, Hao Zhang

**Affiliations:** 1 Lei Ma, Master of Medicine. Department of Anesthesiology and Perioperative Medicine, The First Affiliated Hospital of Xi’an Jiaotong University Xi’an, 710061, China; 2 Hao Zhang, PhD. Department of Surgical oncology, The First Affiliated Hospital of Xi’an Jiaotong University Xi’an, 710061, China

**Keywords:** Ultrasound-Mediated, Intestinal Function Recovery, Lower Gastrointestinal Malignant Tumor, machine learning algorithms

## Abstract

**Objectives:**

In this paper, machine learning algorithms was used to explore the application value of ultrasound contrast in the early evaluation of neoadjuvant chemotherapy in patients with gastrointestinal malignant liver metastases, and analyzes the effect of sports nursing methods on intestinal function recovery.

**Methods:**

Forty-seven patients with gastrointestinal malignancies were divided into 25 patients (combined chemotherapy group) and 22 cases (chemotherapy group) from April 2018 to April 2019. Two groups of patients were treated with CEUS. The effective lesion patients and invalid quantitative parameters were compared between the two groups before and after treatment, and the postoperative routine nursing was implemented.

**Results:**

Chemotherapy group effective in 18 cases, accounting for 81.82%; 4 cases, 18.18%. Combination chemotherapy patients 21 cases, accounting for 84.00%; 4 cases, accounting for 16.00%.

**Conclusion:**

Based on early is important to assess the efficacy of neoadjuvant chemotherapy in patients with liver metastases peak intensity ultrasound contrast parameters of the machine learning algorithms malignant tumors in the gastrointestinal tract, post-operative care movement helps to restore bowel function.

## INTRODUCTION

Measurement of diseases by ultrasound can help doctors judge the disease information in two-dimensional images of human organs.^1^-^3^ In addition, images need to be preprocessed before ultrasound images are used to judge patient information. A lot of effective information and data can be obtained through the processing, and these data can assist doctors to judge the condition. Machine learning algorithm can mine data rules from existing data, and then use the model to carry out prediction research on other data types.^4^-^5^ Computer vision based on machine learning has been widely applied in the field of medical images, such as enhancement method, support vector machine, and principal component analysis.^6^-^8^ Ultrasound image processing by machine learning can be used for auxiliary diagnosis of diseases, which can significantly improve the clinical diagnosis effect of diseases.^9^

Therefore, this study aimed to explore the value of CEUS parameters in evaluating and predicting the efficacy of neoadjuvant chemotherapy before and after chemotherapy in patients with gastrointestinal malignant liver metastasis, and laid a clinical foundation for early treatment planning and improvement of prognosis. Moreover, the postoperative recovery of the elderly patients with digestive tract tumor was also analyzed.

## METHODS

Forty-seven patients with gastrointestinal malignant tumors treated in our hospital from April 2018 to April 2019 were selected, including 26 male and 21 female, aged 35 to 69 years, average age (49.25±12.36) years old. The tumor diameter was between 1.6 and 10.8 cm, with an average diameter of (4.86±1.05) cm; 12 cases of gastric cancer, 24 cases of colon cancer, and 11 cases of rectal cancer.

### Inclusion criteria

1) with pathology and/or cytology confirmed malignancy in patients with liver metastases to the gastrointestinal tract; 2) with pre-neoadjuvant chemotherapy; 3) without severe systemic vital organ dysfunction; 4) with expected survival time of 3 months or more.

### Exclusion criteria

1) a history of drug allergy history of chemotherapy; 2) a previous history of mental illness; 3) the Systemic Acquired immune diseases. Patients were divided into 22 cases of chemotherapy and combination chemotherapy in 25 patients, comparing two groups of patients at an average age of male to female ratio, mean tumor diameter and tumor type, etc., the difference was not statistically significant (P> 0.05).

### Treatment Plan

Patients in the chemotherapy group used FOLFOX chemotherapy regimen (12 cycles), namely oxaliplatin 85mg/m2dl, calcium folinate 400mg/m2dl, 5-fluorouracil 400mg/m2bolus, 2400mg/m2civ46h, repeated every two weeks. Patients in the combined chemotherapy group used bevacizumab combined with FOLFOX chemotherapy regimen (12 cycles), that is, combined with bevacizumab 7.5 mg/m^2^ based on the chemotherapy group.

### Inspection Method

Two physicians with more than three years of experience in ultrasound contrast imaging performed liver ultrasound contrast examinations one day before chemotherapy, 14 days after chemotherapy, 28 days after chemotherapy, and 42 days after chemotherapy. The color Doppler ultrasound diagnostic apparatus uses Italian Yum Mylab90, the probe: CA431, the frequency is 2.0~6.0MHz, the frame frequency is 10-20 frames/s, the mechanical index (MI) is 0.06~0.1, and the corresponding contrast pulse sequence is used Imaging technology. For the same patient, all the parameters of the ultrasound contrast examination before and after chemotherapy remain unchanged.

### Nursing Methods

Perform routine postoperative care for the elderly patients with gastrointestinal tumor surgery in the control group. The patients in the observation group joined the mode of early postoperative exercise care based on routine care. Specific measures are psychological guidance, early postoperative exercise care and two to four days of postoperative care. The responsible nurse can guide and assist the elderly gastrointestinal cancer patients to do some simple rehabilitation exercises, such as walking slowly, standing for a short time, and simple Tai Chi.

### Quantitative analysis of ultrasound contrast

Ultrasonic contrast quantitative analysis software Dynamic Contrast Dynamic Software, representative of the depth of the lesion to select ROI same region of interest (ROI) and normal liver tissue, and by the time - quantitative analysis of tumor hemodynamics intensity curve (TIC). Wherein PI is the highest value of the ultrasound signal intensity, TTP is the initial time to enhance the peak intensity, MTT is the time required to reach half of the peak intensity.

### Principles of machine learning algorithms

In the learning process of the neural network, the appropriate learning function is selected according to the learning samples and different stages of learning to improve the learning speed. The learning function of the neural network can be expressed as


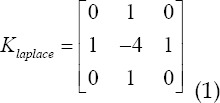


*g(yik)* in different stages of neural network learning, you can take cos(*y*),cosh(*y*),1-*y*^2^ ,tanh(*y*),sin(*y*),*e^-y^* etc. as needed. Through research, it is found that when the expected output value of neuron I in input mode k is 1 and the actual output value is 0, selecting 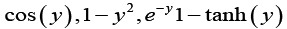 as the learning function can obtain a high convergence speed. The optimization of multi-objective neural network structure is from multiple objectives the function starts (maybe multiple targets are mutually constrained), and the structure of the neural network is optimized through the learning of the neural network to form many specific functional areas (functional nuclei). Specifically, it is to minimize the multi-objective error energy Function E:


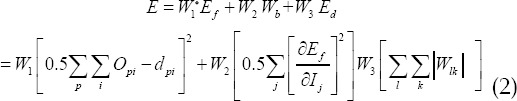


Where *O_pi_* is the actual output of the I node of the output layer in the p input mode, *d_pi_* is the expected output of the I node of the output layer in the p input mode, *E_ljf_* is the derivative of the j node of the input layer, and *W_lk_* is the l The connection weight between the node and the k node. For the convenience of description, this section only discusses the structure of single-layer and multi-layer neural networks. For single-layer neural networks, the Hebb rule can be used as the learning rule of the network, which is.


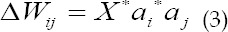


Where X is the learning rate, *a_i_* is the activation value of neuron I, *a_j_* is the activation value of neuron j, and Δ*W_ij_* is the amount of change in the connection weight between neuron I and neuron j. Through learning, neural networks learn from a large number of examples Acquire knowledge and exist in the structure of the neural network in the form of distribution. For multi-layer neural networks, the neural network is trained using the back propagation (BP) algorithm, that is


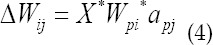


When neuron j is the output neuron,


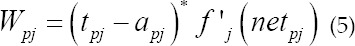


When neuron j is an implicit neuron,


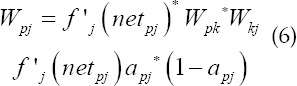


Where Δ*W_ij_* is the amount of change in the connection weight between neuron I and neuron j, X is the learning rate. For the learning of a single-layer neural network, first find *W_kj_* after learning to make it satisfy


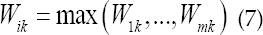


### Evaluation of efficacy

With reference to the amendments made by the National Association of Liver Disease Research (AASLD) for liver cancer, the mRECIST standard was selected to evaluate the clinical treatment effect of patients. Based on the size of the lesion and the extent of the enhancement range, the clinical efficacy includes the following four types: 1) complete remission (CR) 2) partial remission (PR) 3) Stable disease (SD) 4) Disease progression (PD): Among them, CR and PR are effective, and SD and PD are ineffective. The changes of the quantitative parameters of the lesions before and after treatment in the two groups of patients were compared and analyzed.

## RESULTS

Eighteen patients in the chemotherapy group were effective, accounting for 81.82%; 4 were ineffective, accounting for 18.18%. Twenty-one patients in the combined chemotherapy group were effective, accounting for 84.00%; 4 were ineffective, accounting for 16.00%. Compared with chemotherapy therapy patients, chemotherapy patients before treatment effective before PI, AUS, TTP and MTT parameters were not statistically significant (P> 0.05); 14 days after treatment, 28 days and 42 days PI significantly increased rate of change of the parameter, the difference was statistically significant (P <0.05), while the rate of change of the parameter AUS, TTP and MTT other difference not statistically significant (P> 0.05), Table-I.

**Table-I:** Comparison of ultrasound contrast parameters before and after treatment in patients in the chemotherapy group.

	*Ultrasound contrast parameters*	*Effective group (n=18)*	*Invalid group (n=4)*
PI (%)	1 day before treatment	78.69±12.39	80.69±15.37
	14th day after chemotherapy (%)	365.12*	186.29
	28th day after chemotherapy (%)	-439.58*	-275.41
	Day 42 after chemotherapy (%)	-303.75*	-185.46
AUS (%)	1 day before treatment	634.89±92.13	586.97±101.24
	14th day after chemotherapy (%)	-64.89	-58.76
	28th day after chemotherapy (%)	76.28	70.59
	Day 42 after chemotherapy (%)	-59.76	-67.85
TTP(s)	1 day before treatment	6.65±1.56	7.19±1.43
	14th day after chemotherapy (%)	47.89	52.49
	28th day after chemotherapy (%)	29.86	39.16
	Day 42 after chemotherapy (%)	43.54	50.71
MTT(s)	1 day before treatment	98.47±18.45	107.98±27.18
	14th day after chemotherapy (%)	50.73	48.52
	28th day after chemotherapy (%)	38.49	43.75
	Day 42 after chemotherapy (%)	48.67	42.19

Invalid compared to patients, chemotherapy patients before treatment effective before PI, AUS, TTP and MTT parameters were not statistically significant in combination with chemotherapy treatment group; 14 days after treatment, 28 days and 42 days parameter PI significantly increased the rate of change, the difference was statistically significant, while the rate of change of the parameter AUS, TTP and MTT other difference not significant, Table-II.

**Table-II:** Comparison of ultrasound contrast parameters before and after treatment in patients in the chemotherapy group.

	*Ultrasound contrast parameters*	*Effective group (n=18)*	*Invalid group (n=4)*
PI (%)	1 day before treatment	81.33±11.86	82.76±14.37
	14th day after chemotherapy (%)	564.81*	-204.58
	28th day after chemotherapy (%)	-674.38	-187.43
	Day 42 after chemotherapy (%)	-754.12	-258.97
AUS (%)	1 day before treatment	621.49±114.68	607.51±119.57
	14th day after chemotherapy (%)	-71.68	-64.19
	28th day after chemotherapy (%)	79.84	80.43
	Day 42 after chemotherapy (%)	-54.69	-61.3
TTP(s)	1 day before treatment	7.09±1.84	7.23±1.56
	14th day after chemotherapy (%)	51.82	54.71
	28th day after chemotherapy (%)	34.59	37.1
	Day 42 after chemotherapy (%)	38.52	46.73
MTT(s)	1 day before treatment	104.58±15.38	108.09±24.71
	14th day after chemotherapy (%)	48.16	52.17
	28th day after chemotherapy (%)	41.85	46.53
	Day 42 after chemotherapy (%)	49.86	50.11

## DISCUSSION

In liver metastasis of gastric cancer, metastasis is mainly carried out by hematogenous, lymphatic, or primary tumor invasion, and the incidence of metastasis is 4%-14%.^10^-^12^ At present, the treatment methods for liver metastasis of gastric cancer mainly include surgery, ablation, interventional therapy, and targeted therapy, among which systematic therapy includes a series of therapeutic measures such as NAC and postoperative chemotherapy.^13^-^15^ NAC therapy is often used to treat patients with advanced cancer. It is effective in removing invisible metastatic cancer cells and reducing tumor size. It is of great significance for disease control in patients with liver metastasis of gastric cancer and reducing postoperative intrahepatic recurrence rate.^16^-^18^ In this work, the therapeutic effect of neoadjuvant chemotherapy on patients with liver metastasis of gastric cancer was compared. The results showed that the change rate of PI was significantly increased in the chemotherapy group at 14, 28, and 42 days after treatment compared with the patients in the chemotherapy group. PI is the echo intensity caused by the peak value of contrast agent, which reflects the degree of fibrosis of gastric cancer lesions.^19^,^20^ In short, neoadjuvant chemotherapy can significantly improve the degree of fibrosis in the focal area of patients with liver metastasis of gastric cancer and reduce the progression of patients. However, the monitoring period of patients after treatment with different treatment methods is short, and the changes of complication and survival time after treatment are lacking. Based on this, we will follow up patients to provide a long-term reference for improving the clinical treatment effect of patients with liver metastasis of gastric cancer.

## CONCLUSIONS

In summary, the peak intensity of ultrasound contrast parameters is of great significance in the early evaluation of neoadjuvant chemotherapy for patients with gastrointestinal malignant liver metastases. For the elderly patients with gastrointestinal tumor surgery, the responsible nurse can adopt the way of early postoperative exercise care to effectively promote the rehabilitation of patients, and the clinical application value is more significant.

### Authors’ Contribution:

**LM:** Conceived the study, literature review, data analysis, drafting the paper.

**HZ:** Takes the responsibility and is accountable for all aspects of the work in ensuring that questions related to the accuracy or integrity of any part of the work are appropriately investigated and resolved.
